# In vivo assessment of dog subcutaneous fat depots by real time ultrasonography and image analysis

**DOI:** 10.1186/1751-0147-57-S1-P9

**Published:** 2015-09-25

**Authors:** Rita Payan-Carreira, Luis Martins, Sónia Miranda, Pedro O Pinto, Severiano R Silva

**Affiliations:** 1 Animal and Veterinary Research Center, CECAV, Universidade de Trás-os-Montes e Alto Douro, Vila Real, Portugal; 2 Escola Universitária Vasco da Gama, Coimbra, Portugal; 3 Hospital Veterinário do Baixo Vouga, Águeda, Portugal

## Introduction

Obesity is the most common nutritional disorder in dogs and a main health and welfare concern worldwide; it links to a shortened lifespan and increased rate of secondary diseases. Surveillance of dog adiposity is a routine practice and is often estimated from body condition scoring (BCS). But BCS is subjective and fairly sensitive, reducing its utility during weight-loss plans. Substitute approaches, simple, cheap and reproducible, are foreseen.

## Objective

This work aimed to assess real time ultrasonography (RTU) usefulness for analysis of sub-cutaneous body fat depots (SCF) in dogs.

## Methods

Twenty-eight dogs were enrolled, representing different sizes (nain-4; small-10; medium-14), weights (BW; 5.2–33.0 kg) and BCS (2-4 in a 5 points scale). RTU images were taken with a multifrequency linear array (at 10 MHz) coupled to a GE scanner, from non-sedated dogs in right lateral recumbency, at five anatomical points: entry of the chest; over the ninth intercostal space; lateral abdominal wall; right inner thigh; and between the third and the fifth lumbar vertebrae. Images were analysed in Image J; means from 3-different locations per image were used to set SCF thickness. Using the JMP program correlation procedure was used to analyse SCF and BCS relationships.

## Results

BW was poorly associated with SCF thickness (r between 0.21, p>0.05, and 0.59, p<0.01), while BCS and SCF were strongly correlated (r between 0.71 and 0.82; p<0.01), particularly for data collected at lumbar and abdominal points.

## Conclusion

Results stress that BW is a poor adiposity predictor and suggest that RTU is a valuable tool to predict dog adiposity.

